# Biomarkers of resistance to immune checkpoint inhibitors in non-small-cell lung cancer: myth or reality?

**DOI:** 10.20517/cdr.2020.14

**Published:** 2020-05-11

**Authors:** Ivan Pourmir, Benoit Gazeau, Hortense de Saint Basile, Elizabeth Fabre

**Affiliations:** ^1^Department of Thoracic oncology, Hôpital européen Georges Pompidou, Paris 75015, France.; ^2^INSERM U970, Université Paris Descartes Sorbonne Paris-Cité, Paris 75015, France.

**Keywords:** Non-small-cell lung cancer, immune checkpoint inhibitors, resistance, predictive biomarkers, diagnostic biomarkers, programmed death ligand-1, tumor mutational burden, circulating tumor DNA

## Abstract

Immune checkpoint inhibitors represent a major therapeutic advance in non-small-cell lung cancer with several approved anti-programmed death-1 and anti-programmed death-L1 immunotherapies. A majority of patients however, will not respond to immune checkpoint inhibitors and display primary resistance while a subset of initially responsive patients will present secondary resistance. Thus, there is a crucial need for biomarkers to enable better prediction and diagnosis, and to overcome such resistance. Along with improvement in the understanding of immune escape, new biomarkers are being developed, including large scale proteomic, genomic and transcriptomic approaches in tumor and blood samples. We review the novel biomarkers that have been investigated in non-small-cell lung cancer and discuss how they can rationalize therapeutic strategies.

## Introduction

Immune checkpoint inhibitors (ICI) such as programmed death-1 (PD-1) and programmed death ligand-1 (PD-L1) inhibitors represent a major breakthrough in the treatment of non-small cell lung cancer (NSCLC). However, only 20%-30% of NSCLC patients respond to anti-PD-1/PD-L1^[1-5]^. There is therefore a crucial need to integrate biomarkers of resistance for a more personalized approach to immunotherapy.

“Primary resistance” is found in patients who have never responded to ICI whereas “secondary/acquired resistance” is defined by tumor progression after prior response^[6]^. The efficacy of ICI relies on the de-repression of immune checkpoints hindering the action of existing anti-tumor lymphocytes. Thus, several steps of the adaptive immune response need to be fulfilled: (1) production by tumor cells of tumor-specific antigens; (2) activation and expansion of T-cells specific for these antigens; (3) migration to and infiltration by effector T-cells of target cells; (4) recognition of target tumor cells; and (5) cytotoxic and immuno-stimulatory activity of the effector T-cells.

Mechanisms of resistance to ICI can impair each step of this immune response^[7]^. We review the existing and potential biomarkers to discuss which could serve as practical tools to predict or diagnose resistance to ICI.

## Resistance biomarkers related to intrinsic factors of tumor cells

Mechanisms of intrinsic resistance include defects in immune recognition, cell signaling, gene expression and DNA damage responses. Emerging biomarkers are developed after considering these different mechanisms of resistance.

### Oncogenic PD-L1 expression

Theoretically, the efficacy of ICI targeting the PD-1/PD-L1 axis relies on the de-repression of effector T-cells inhibited by the binding of PD-L1 on their membranes receptors. PD-L1 expression is currently the only validated biomarker in patients. Indeed, immunohistochemistry (IHC) staining of PD-L1 in tumor cells or immune cells is more frequently observed in responders to anti-PD-1/PD-L1 monoclonal antibody therapies^[2-5,8]^. However, even when a tumor displays > 50% PD-L1 staining, half of NSCLC patients still have primary resistance to first line Pembrolizumab^[8]^. Conversely, clinical benefit is found in 10% of patients negative for PD-L1^[2,9]^.

Historically, the development of ICI in NSCLC has been based on the PD-L1 status of tumor cells, specifically, via a tumor proportion score^[8]^, which is the percentage of viable tumor cells showing partial or complete membrane staining at any intensity. Given the rising role of PD-L1 expression on non-tumor cells, the use of a combined positivity score has been developed by taking into account the number of PD-L1 staining cells (tumor cells, lymphocytes, macrophages^[10]^). However, PD-L1 expression, whether on tumor cells or immune cells, still appears insufficient in predicting resistance. Other PD-1 ligands though, may be relevant to the clinical activity of ICI. The prevalence of programmed cell death-ligand 2 (PD-L2), the other known ligand of PD-1, has been investigated^[11,12]^. PD-L2 expression was correlated with treatment response using a cut-off value of 50% expression in tumor cells in NSCLC^[11]^.

Pre-analytical, analytical, and post-analytical aspects of PD-L1 IHC testing such as specimen type, size of samples, diversity of IHC antibodies, and lack of standardization of positivity cut-offs can all influence PD-L1 results^[13,14]^. Moreover, the intra-tumor heterogeneity of PD-L1 expression^[15]^ must be taken into account^[8]^ in anticancer treatment^[16]^.

### Lack of tumor immunogenicity

#### Low tumor mutational burden, insufficient neo-antigens

Under certain conditions, mutations occurring in the genetic material of tumors can lead to the production of abnormal proteins, which can then be processed by the proteasome and antigen presenting machinery, resulting eventually in the presentation of immunogenic tumor specific neo-antigens^[17]^. Thus, it has been hypothesized that a higher frequency of non-synonymous mutations in tumor cells, the so called “tumor mutational burden (TMB)”, should result in a higher rate of neo-antigen production and probability of triggering an adaptive immune response.

In NSCLC, a high, non-synonymous mutation burden is defined by a threshold superior to 178 mutations/sample after whole exome sequencing (WES) and has been correlated to response, durable clinical benefit and progression-free survival. Conversely, a low TMB predicts poor efficacy in NSCLC patient cohorts and can be considered as a marker of primary resistance to ICI^[18]^. The association between TMB and response to ICI was demonstrated globally in a meta-analysis across 27 tumor types, including NSCLC^[19]^.

Multiple gene panels have also been used to evaluate the TMB in a more feasible way than WES with good accuracy^[20]^. In Checkmate 227, a next generation sequencing (NGS) panel of 324 genes^[21]^ was assessed by the FoundationOne CDx assay in NSCLC patients treated with the combination of Ipilimumab and Nivolumab. A good correlation between progression-free survival and TMB status was found using a threshold defined by 10 mutations/megabase.

TMB data have also been extracted from blood samples. The advantages of this approach are the convenience of blood sampling and a more global estimate of TMB given heterogeneity concerns in tissue samples^[22]^. Gandara *et al.*^[22]^ demonstrated the validity of TMB assessment in blood samples (bTMB) for predicting the response of metastatic NSCLC patients to Atezolizumab. The technique relied on the association of NGS of a large portion of cell-free DNA, allowing the identification and quantification of abnormal tumor sequences related to circulating tumoral DNA. In the MYSTIC study, metastatic NSCLC patients displaying a low bTMB (defined as < 16 mutations/megabase in the Guardant Health Omni assay, a NGS panel of 500 genes^[23]^) had lower overall survival under Durvalumab + Tremelimumab treatment compared to standard chemotherapy^[24]^.

However, the CheckMate227 study failed to demonstrate correlation between overall survival and TMB and this revived debates about diagnostic techniques^[25,26]^. Beyond the crucial need to standardize practices (pre-analytical parameters, thresholds, type of samples or sequencing methods), it has also been hypothesized that the TMB may not be a sufficient marker for predicting primary resistance to ICI^[27,28]^.

One should also keep in mind that mutations occurring in non-exonic regions of tumor DNA can lead to abnormal mRNA and peptide sequences, particularly via alternative splicing, thus generating potential neo-antigens that would not be predicted using the methods described above^[29]^. A refinement of TMB analysis could be neo-antigen burden assessment since only mutations resulting in the production of these immunogenic antigens would be relevant for predicting the existence of an adaptive anti-tumor response. Such an approach has been used in NSCLC via bioinformatics tools to enable calculation of neo-antigen load from genomic and transcriptomic data^[30]^. Indeed, a low neo-antigen burden has been associated with poor treatment response to anti-PD-1^[8,30]^. McGranahan *et al.*^[30]^ focused on the importance of the tumoral distribution of neo-antigenes and showed that clonal neo-antigens predominant in responders are more immunogenic than sub-clonal antigens.

#### Loss of neo-antigens/immuno-editing

Conversely, several mechanisms including immunoediting, can potentially lead to the loss of neo-antigens that enable primary responses to ICI, such that tumors develop secondary resistance. This phenomenon seems to be facilitated by the existence of intra-tumoral genomic heterogeneity, both at baseline and after PD-1 blockade^[31]^. Immunoediting has also been observed in NSCLC by dynamic blood sampling along anti-PD-1 therapy. The decrease in the number of circulating anti-tumor T-ell clones (see below “Clonality of tumor-specific T-cells”) correlated with secondary resistance, suggesting a decrease in the number of neo-antigens stimulating the anti-tumor immune response^[32]^.

#### Oncogenic addiction

Phase III trials have failed to demonstrate the efficacy of PD-1/PD-L1 monoclonal antibody in patients with EGFR and ALK mutations, which suggests the co-expression of inhibitory receptors or T-cell interactions with immunosuppressive cells^[3-5]^. EGFR results were summarized in a meta-analysis^[33]^. A retrospective study on patients receiving ICI monotherapy for advanced NSCLC with at least one oncogenic driver alteration (EGFR, BRAF, MET, HER2, ALK, RET, or ROS1), showed lower clinical activity compared with the KRAS group and the lack of response in cases of rearrangement^[34]^. In the subset of KRAS-mutated lung adenocarcinomas, co-mutation of the tumor suppressor gene STK11 is associated with a lack of response to ICI^[35]^.

#### Antigen presentation defect in tumor cells: loss of human leukocyte antigen expression

Events altering antigen presentation by the major histocompatibility class I complex (MHC-I) occur at a different level and are believed to prevent ICI efficacy. These include genetic, transcriptional, post-transcriptional and epigenetic mechanisms^[36]^. McGranahan *et al.*^[37]^ applied a specifically designed computational method on DNA sequencing data from the HLA locus of NSCLC. They found a 40% prevalence of loss of heterozygosity in HLA class I alleles, impairing neo-antigen presentation by tumor cells. HLA loss of heterozygosity also seemed to be acquired heterogeneously in tumor sub-clones, as an adaptive mechanism in immune escape. This suggests that the assessment of HLA loss of heterozygosity could predict resistance in spite of a favorable tumor microenvironment and/or high TMB.

#### Impaired cytotoxicity of effector T-cells

Decreased interferon-gamma signaling. Interferon-gamma (IFN-γ) is produced by CD8^+^ T-cells upon activation, as well as other inflammatory cells, and is a major effector of anti-tumor activity by enhancing the antigen-presentation machinery and having strong immune stimulatory properties. Defects in the IFN-γ signaling pathway have been identified as a major mechanism of resistance^[38]^ to PD-1 and CTLA-4 blockade^[39]^. It has been shown that genetic alterations of the Janus kinase/signal transducers and activators of transcription pathway^[38,40]^ can lead to secondarily PD-L1 negative tumor cells with altered antigen presentation. Therefore, transcriptomic analyses have been performed in order to identify a gene expression signature predictive of ICI efficacy. Panels of IFN-γ signature genes have allowed prediction of responses of NSCLC patients to anti-PD-1/PD-L1^[41,42]^. A decrease in the level of expression of these genes could be interpreted as a lack of CD8^+^ activity. Two different IFN-γ signatures including respectively 8 and 4 genes showed a significant association with overall survival in NSCLC patients treated by anti-PD-L1^[41,43]^. However, in lung and other types of cancer, results are contradictory^[44-46]^. It must be specified that IFN-γ is also produced by a variety of inflammatory cells, leading to a transcriptional elevation of *IFN-*γ genes and therefore, some imprecision in result interpretation. On the other hand, prolonged IFN-γ stimulation leads to the expression of PD-L1, and thus, immune escape.

Production of immunosuppressive metabolites. The enzyme indole 2,3-dioxygenase (IDO) has an immunosuppressive activity in tumors and are associated with ICI resistance^[47,48]^. IDO expression, assessed by IHC, has commonly been found in NSCLC, notably in PDL-1 positive tumors^[49]^. Concentrations of these metabolites were measured with liquid chromatography and mass spectrometry^[50]^ and a higher kynurenine to tryptophan ratio and quinolinic acid at baseline, resulting from IDO metabolic activity, was found in plasma samples of NSCLC patients with early progression under Nivolumab treatment. To overcome this putative mechanism of resistance to ICI, the combination of ICI with IDO specific inhibitors are currently being evaluated in pre-clinical and human studies.

## Resistance biomarkers related to immune reaction and host factors

### Insufficiency of tumor-specific T-cell activation

#### Impaired T-cell trafficking to tumor cells and tumor infiltration

Chemokines regulate the infiltration of different immune cell subsets into tumors. As such, these molecules affect tumor immunity and can influence therapeutic outcomes in patients^[51]^. High levels of intra-tumoral chemokines such as CCL5, CXCL9, CXCL10 enhance the recruitment of T-cells into the tumor^[52]^ and preclinical studies in mice showed that infiltrating anti-tumor CD8^+^ T-cells are required for clinical response to anti-PD-1 treatment^[53]^. Similar studies in humans have shown that the pre-existing CD8^+^ T-cells in the tumor microenvironment correlate with response to anti-PD-1/PD-L1 therapies in various cancer types, including NSCLC^[44,54,55]^. Thus, the lack or paucity of T-cell infiltration of tumors characterizes the more general concept of “hot” and “cold” tumors with regard to the immune micro-environment^[56]^. The exclusion of T-cells involves multiple mechanisms such as the inhibition of T-cells attracting chemokines. *In vivo*, transforming growth factor beta, a multipotent immunosuppressive cytokine, suppresses CD8^+^ T-cell expression of CXCR3 and limits their trafficking into tumors^[57]^.

The presence of intra-tumoral CD8^+^ T-cells is not always associated with clinical benefit however^[44]^. Therefore, there is growing interest to improve the identification of subsets of tumor-infiltrating lymphocytes (TILs) and tumor-specific lymphocytes. Herbst *et al.*^[44]^ propose that PD1 expressing CD8^+^ T-cells, better select for patients responding to anti-PD-144. Clarke *et al.*^[58]^ have reported that the number of tumor-resident CD8^+^ T-cells at baseline, and a CD103^+^CD49^+^CD69^+^ subset of TILs, are more predictive of response to anti-PD-1 therapy than total CD8^+^ T-cells in NSCLC and could be a potential marker of primary resistance to anti-PD-1 monotherapy.

#### Clonality of tumor-specific T-cells

It is now admitted that a restricted T-cell receptor sequence reflects the accumulation of TILs specific for a restricted number of tumor antigens at the invasive margins of the tumor. The sequencing of the T-cell receptor β-chain repertoire of TILs revealed that patients with a low “clonality” are more likely to respond to immunotherapy in several types of cancers^[54,59,60]^ using median clonality as a threshold.

#### Impaired expansion of T-cells

Expansion of anti-tumor specific T-cells following ICI therapy is a dynamic marker of treatment response and has recently been found to correlate with primary resistance to PD-1/PD-L1 blockade in metastatic NSCLC^[32]^. However, the direct isolation and quantification of tumor-specific lymphocytes required a complex and customized process, given that the neo-antigen repertoire is unique. For this reason, indirect markers for measuring variations of this cell population could be more convenient.

### Alternative immune checkpoints

The presence of CD8^+^ is not sufficient as they must be functional. Apart from PD-1, the expression of many alternative immune checkpoints can be found in NSCLC at baseline, or after prior blockade of the classic immune checkpoint pathways and are likely to contribute to primary or secondary resistance to ICI blockade^[61]^. The increased expression of TIM-3 (T-cell immunoglobulin and mucin domain 3), LAG-3 (lymphocyte activation gene 3), and BTLA (B and T lymphocyte attenuator) on CD8^+^ TILs are associated with adaptive resistance to anti-PD-1 in NSCLC^[62-65]^.

Fluorescence measurements of various immune checkpoints were also performed using blood samples. Non-responding patients showed a stability of fluorescence levels before and after the first cycle whereas responding patients displayed a dramatic decrease of CTLA-4, GITR (glucocorticoid-induced tumor necrosis factor receptor), and OX40 (CD134) expression on CD4^+^ and natural killers cells after the second cycle of immunotherapy^[66]^.

### Exhaustion of T-cells

Exhausted T-cells demonstrate altered anti-tumor function and decreased re-invigoration potential under ICI, as well as an impaired capacity to generate T memory cells^[7,67,68]^. Terminally exhausted TILs are identified by the pattern of membrane markers with a high expression of CD38, CD101, and CD30 and low expression of CD5^[69]^. The co-expression of multiple immune checkpoint receptors^[63]^ has been associated with a severely exhausted state and failure to rescue function with Nivolumab *in vitro*. These tumor-infiltrating but exhausted CD8^+^ cells could allow us to predict which patients would not respond to ICI because of terminal exhaustion^[69]^.

### Immunosuppressive cells

Under certain conditions, the microenvironment favors the recruitment of immunosuppressive cells such as regulatory T-cells (Treg) and myeloid-derived suppressor cells, which induce the production of adenosine, an immunosuppressive molecule. The detection and quantification of several immunosuppressive cells within the tumor micro-environment is ongoing to assess their ability to predict ICI resistanc^[7,70]^. The quantification of Treg and myeloid-derived suppressor cells has also been analyzed to predict resistance to ICI in melanoma and gastric cancer^[71-73]^. No direct association has yet to be observed in NSCLC however.

### Resistance biomarkers related to the host: microbiota

A strong immuno-modulatory effect of gut microbiota in the context of cancer has recently been reported. In the feces of NSCLC patients, the baseline paucity of several commensal bacteria species, after evaluation with a shotgun metagenomic analysis, has been associated with poor response to anti-PD-1 treatment. This could be reversed by fecal transplantation in a mouse model^[74]^. As for now, there is still a lack of congruence between the results of different studies correlating gut microbiota profiles to response to immune therapy to determine precisely which would be predictive of primary resistance, and would need to be modified for subsequent treatment^[75]^.

## Discussion

Resistance biomarkers are necessary at each stage of disease to define therapeutic strategies. Prior to the initiation of treatment, they are expected to rationalize the choice between monotherapy and combination therapy. During treatment, they are also needed to distinguish between radiological and pseudo-progression, and in cases of progression they are essential for personalized treatment.

From a short-term point of view, as the majority of patients currently eligible for ICI do not respond, such biomarkers would allow early cessation of and switching to another treatment. Notably, in the case of localized NSCLC, this would allow neoadjuvant immunotherapy without losing the opportunity of proceeding to surgery. In oligo-metastatic patients, the kinetics of sensitive biomarkers would also help in making decisions on complementary local treatment for complete cytoreduction. Lastly, an undetectable or dramatic decrease in systemic levels of biomarkers would allow suspending ICI safely in case of serious adverse events or persistent complete response.

Until now, the only validated marker to guide ICI prescription in NSCLC, outside of clinical trials, remains PD-L1 [Table 1]. As discussed above, this indicator is far from sufficient and still need to be refined and harmonized.

**Table 1:** Predictive biomarkers for resistance to immune checkpoint inhibitors in NSCLC

BioMarker	Treatment	Population	Methods	Findings associated with resistance	Ref.
TMB	Anti-PD-/PD-L1; Anti-PD-1/PD-L1 + anti-CTLA-4	Advanced NSCLC	WES; NGS gene panels; Tissue and blood samples	Low mutational burden correlates with poor response, reduced OS and PFS	[18,20,22,27]
Tumor neoantigen burden	Pembro	LUAD	WES, tissue samples	Low neo-antigen burden correlates with poor OS	[30]
Intra-tumor neoantigen heterogeneity	Pembro	Early stage NSCLC	WES; resected NSCLC	High intra-tumor neo-antigen heterogeneity correlates with poor OS	[30]
TILs	Pembro; Nivo	Advanced NSCLC	Anti-CD4; anti-CD8 IHC staining; tissue samples	Low CD8^+^ T-cells density and CD8^+^/CD4^+^ ratio correlate with poor response	[44]
PDL-1	Anti-PD-1/PD-L1	NSCLC	Anti-PD-L1 IHC staining of tumor cells, immune cells; PDL1 mRNA; tissue samples	Low PD-L1 density expression predicts poor response, PFS and OS	[2,8,43]
Alternative IC	Anti-PD-1/PD-L1	NSCLC	Multiparametric FACS detecting alternative IC on CD8^+^ TILs at baseline/after ICI	Co-expression of alternative IC is associated with primary and secondary resistance	[61,62]
IFN-ɣ	Pembro; Nivo; Durva	Advanced NCLC	Gene panels transcription; tumor sample	Decreased IFN-γ expression correlates with poor response and OS	[41,42]
IDO	Nivo	Advanced NCLC	Liquid chromatography and mass spectrometry; plasma samples at baseline	High kynurenine/tryptophan ratio and quinolinic acid level associated with poor response	[50]
Microbiota	Nivo	Advanced NCLC	Shotgun metagenomic analysis of feces at baseline	Low metagenomic species richness and distinct profiles correlate with poor response	[74]
Circulating tumor-reactive CD8^+^ clones	Pembro; Nivo	Metastatic NSCLC	FACS on blood samples; TCR sequencing	Decreased number of circulating clones correlates with secondary resistance	[32]
Alternative immune checkpoints	Pembro; Nivo	Advanced NSCLC	CTLA-4, GITR, and OX40 fluorescence on CD4^+^ and NK cells; blood samples	Stability of CTLA-4, GITR, and OX40 fluorescence after 1st cycle correlates with primary resistance	[66]

IC: immune checkpoints; NSCLC: non-small cell lung cancer; NK: natural killers; GITR: glucocorticoid-induced tumor necrosis factor receptor; TCR: T-cell receptor; IDO: indole 2,3-dioxygenase; OS: overall survival; ICI: immune checkpoint inhibitors; IFN-γ: interferon-gamma; PD-L1: programmed death ligand-1; TILs: tumor-infiltrating lymphocytes; PFS: progression-free survival; IHC: immunohistochemistry; WES: whole exome sequencing; NGS: next generation sequencing; TMB: tumor mutational burden; FACS: fluorescence-activated cell sorting; LUAD: lung adenocarcinoma

Currently, TMB appears as one of the potential biomarkers likely to guide future ICI prescriptions in the near future. The inter-individual diversity of alterations makes comprehensive approaches such as WES or analysis of large panels of genes increasingly attractive^[21]^. The speed of technical improvement in this field renders short-term routine exploitation realistic and should warrant consideration to spare obtaining sufficiently high amounts of tissue or blood from our current patients.

At the histopathological level, the increasing number of relevant cellular types to identify, coupled with the necessity of analyzing their spatial relationships, should lead us to multi-parametric *in situ* imaging methods such as MIBI^[76]^ or CODEX^[77]^. Multiplexed IHC represents a promising approach to analyze immune composition and define precisely CD8^+^ cell subpopulations, location and functionality.

In addition to the complexity of their immune composition, biomarkers must integrate immune changes during treatment. As an example, in a longitudinal study from Chen *et al*.^[78]^, sequential gene expression profiling of melanoma found no baseline difference between responders and non-responders to anti-PD-1 therapy, but hundreds of genes were then differentially expressed in early on-treatment biopsies. Therefore, repeated samplings represent a promising solution to assess the dynamic nature of treatment resistance and will probably remain key for treatment adaptation in patients responding to ICI. However, blood-based analysis represents a growing field of interest to achieve immune-monitoring. Other approaches using bronchoalveolar lavage fluid are in development to evaluate the local immune status at the site of lung cancer. This procedure may be performed during lung cancer diagnosis and repeated during therapy^[79]^.

The variety of biomarkers and resistance mechanisms discussed in this review [Table 1], and their cooccurrence at a single-patient-level, in addition to the growing number of available immunotherapies, emphasizes the necessity of including a much larger number of biological parameters in our strategies. This implies a shift in the scale of biological analyzes and techniques that are presently in use. Immunotherapy monitoring will certainly need both tissue and blood sampling and the combination of several biomarkers. Beside PD-L, other biomarkers are beginning to emerge: specific immune cell sub-populations analyzed by multiplexed IHC, plasmatic TMB, circulating immune cells and their antigenic repertoire.

Current advances including molecular profiling and specific tumor-associated immune characterization, allow us to hypothesize the following strategy:

1. Tissue and blood sampling with pathological and genomic analysis, all at baseline, in order to assess tumor immunogenicity and potential mechanisms of primary resistance;
2. Determination of first line therapy: ICI monotherapy or combination;
3. Early assessment of treatment efficacy and detection of primary resistance;
4. Second line treatment or addition of supplementary antineoplastic compounds in case of primary resistance;
5. Treatment pause or discontinuation when prolonged remission is achieved;
6. Suspicion of clinical and radiological relapse and confirmation with systemic biomarker;
7. Determination of optimal second line immunotherapy.

Several pre-requisite have yet to be fulfilled however, for new potential biomarkers to be routinely applied. The standardization of pre-analytic variables is also required^[80]^, as the definition of precise thresholds varies.

Evaluation of this exciting progress must also integrate the cost of innovation. The escalating health expenditure in cancer care in Western countries is of particular concern and the cost of performing biological tests must be taken into account. This requires an assessment of the overall spending related to cancer cases and one should not simply sum up the costs for the tests alone. Efficient biomarkers for monitoring disease status would also allow treatment discontinuation in cases of remission and subsequent economics. For example, the cost of WES for one individual has reached a lower order of magnitude than the cost for a single cycle of ICI therapy^[81]^.

## Conclusion

The complexity of the immune system requires the combination of resistance biomarkers to define therapeutic strategies in NSCLC. A global approach integrating both immune and tumor-related parameters is needed. Novel composite and dynamic biomarkers of immune evasion are emerging to guide personalized immunotherapies. The simultaneous analysis of PD-L1 expression, specific anti-tumoral CD8^+^ infiltration and TMB, seems a highly promising approach.

## Declarations

### Copyright

© The Author(s) 2020.
